# Social Media as a Tool for Consumer Engagement in Hospital Quality Improvement and Service Design: Barriers and Enablers for Implementation

**DOI:** 10.34172/ijhpm.2021.151

**Published:** 2021-11-03

**Authors:** Louisa Walsh, Nerida Hyett, Jayne Howley, Nicole Juniper, Chi Li, Belinda MacLeod-Smith, Sophie Rodier, Sophie Hill

**Affiliations:** ^1^Centre for Health Communication and Participation, La Trobe University, Bundoora, VIC, Australia.; ^2^La Trobe Rural Health School, La Trobe University, Bendigo, VIC, Australia.; ^3^Independent Researcher, Melbourne, VIC, Australia.; ^4^Orygen, Parkville, VIC, Australia.; ^5^Albury Woonga Health, Wodonga, VIC, Australia.; ^6^Safer Care Victoria, Melbourne, VIC, Australia.; ^7^Northern Health, Bundoora, Australia.

**Keywords:** Social Medi, Australia, Service Design, Quality Improvement, Consumer Engagement, Patient Involvement

## Abstract

**Background:** Social media can be used to engage consumers in hospital service design and quality improvement (QI) activities, however its uptake may be limited by a lack of guidance to support implementation. This article presents the perceived barriers and enablers in using social media for consumer engagement derived from an interview study with public hospital stakeholders.

**Methods:** Semi-structured interviews with 26 Australian hospital service providers and consumer representatives. Data were analysed using a deductive content analysis method.

**Results:** Data were collected between October 2019 and April 2020. Facebook was the platform most commonly used for consumer engagement activities. Barriers and enablers to social media-based consumer engagement were identified. The barrier themes were (1) fears and concerns; (2) lack of skills and resources for social media engagement; (3) lack of organisational processes and support; and (4) problems with social media platforms and the changing social media landscape. The enabler themes were: (1) hospitals facilitating access and use; (2) making discussions safe; (3) cultivating a social media community; and (4) building on success.

**Conclusion:** Using social media to facilitate consumer engagement in hospital service design and QI activities is feasible and acceptable to service providers and consumers. Hospitals and their executives can create a supportive environment for social media-based engagement activities through developing clear governance systems and providing training and support to all users. Consumers need to be involved in co-designing social media-based activities and determining which forms of engagement are accessible and acceptable. For some consumers and service providers, barriers such as a lack of resources and distrust of social media companies might mean that social media-based engagement will be less acceptable for them. Because of this it is important that hospitals provide complementary methods of engagement (eg, face-to-face) alongside social media-based methods.

## Background

**Key Messages**

** Implications for policy makers**

- Consumer engagement in service design and quality improvement (QI) is a policy or accreditation requirement in many countries. Stimulus and support will continue to be needed by hospitals and this should encompass guidance and support on using social media. Because the technology and behaviours associated with social media are also changing, the benefits of social media for broader two-way communication will require work to be realised.
- Some identified barriers to social media-based engagement, such as ensuring universal and equitable access to high-speed and low-cost internet services, can only be addressed at the policy level.
- Learning from the experience of others, and sharing successful social media-based consumer engagement projects, were important enablers for uptake of social media-based consumer engagement methods. Government health departments could provide opportunities for hospitals and health services to share their successes and experience to build local knowledge, expertise and confidence around social media-based engagement methods.

** Implications for the public**

This study may provide members of the public with new strategies for engaging with their hospitals and health services if they want to become involved in service design or improvement activities. The findings could also be used by members of the public or consumer groups to advocate for the uptake of social media-based engagement methods in their hospital or health service.

Social media is becoming a feature of health and healthcare. The collaborative communication, user-generated content and networking which define social media^1,2^ has seen it used in health for sharing information,^3-7^ peer-to-peer connection for consumers and service providers,^5-10^ and organisational data gathering.^4,6,10^ A recent scoping review by members of this author team also demonstrated that social media has been used as a tool for stakeholder engagement in health service design and quality improvement (QI).^11^ Social media was used to engage stakeholders through consultative activities, collectivisation and advocacy activities, development of learning networks for people working on QI projects and as virtual settings for collaborative project work.^11^ Patient experience data which informed service design and QI activities was also gathered through social media.^11^ The majority of activities were conducted in high-income countries.^11^

 Compared to more typical methods of consumer engagement (eg, interviews, focus groups, consumer representation on committees, surveys^12,13^) social media has the potential to increase the numbers of people reached by engagement activities, engage more diverse audiences, increase the speed of engagement, and reduce barriers to engagement associated with face-to-face activities.^10,11,14,15^ Because social media can reach audiences different to those reached by typical engagement methods, it may help grow the pool of people engaged in health service design and QI, and expand or re-imagine engagement strategies beyond the overreliance on ‘career consumers.’^16^ Social media-based engagement can also allow consumer representatives, patients and families to connect more easily to organise and advocate for change,^15^ which could help overcome potential disconnection between patient populations and their representatives.^17,18^

 The use of social media for consumer engagement is not without risks. Engagement methods characterised by relationship building between consumers and service providers (such as co-design) might be more difficult via social media,^11,15^ and the public nature of interactions on some social media platforms could expose both individuals and organisations to harms such as bullying, harassment and trolling.^11,15^ Concerns about these risks, and a lack of understanding about ways to manage or mitigate them, may stymie efforts to further develop social media as a medium for engagement.^15^

 To better understand and inform the implementation of social media-based consumer engagement in public hospitals, we explored the following research question:

 ‘What experiences and opinions do public hospital stakeholders have around the use of social media as a tool to facilitate consumer engagement in hospital service design and QI?’

 To answer this question, Australian public hospital stakeholders were interviewed. People in Australia have high levels of internet and social media use (89% of the population use the internet,^20^ 80% use social media^21^), but also experience low health literacy (only 40% of adults are estimated to have sufficient health literacy to effectively manage their health^22^). The uptake of social media by hospitals for a range of functions – including consumer engagement – may be lower than other high-income countries.^11^ Australian hospitals have also identified a need for guidance^11^ around the use of social media for consumer engagement.^19^ This context means that Australian public hospitals provide a suitable environment in which to explore both positive and negative aspects of social media-based consumer engagement. Additionally, most previously published research into social media-based engagement in health service design and improvement has presented case studies of single activities with a focus on the process of the activity,^11^ rather the experience of consumers and service providers of using social media as an engagement tool we explored through our research.

 This article presents the findings from this research relating to the barriers and enablers for social media-based consumer engagement described by the participants. These findings might assist implementers, both in Australia and internationally, in their planning of social media-based consumer engagement. Findings relating to risks and barriers of social media-based engagement have been presented in a previous article arising from this study.^15^

 Finally, we are using the term ‘consumer engagement’ in this article to describe involvement of users, or potential users, of health services in their design and improvement.^23^ The term ‘consumer representative’ in this article refers to “a person who provides a consumer perspective, contributes consumer experiences, advocates for the interests of current and potential health service users, and takes part in decision-making processes”(p. 75)^23^ and is currently engaged in a consumer representative role within a public hospital. These terms are commonly used in Australian public hospitals and government health departments,^23^ but we appreciate there is long-standing lack of consensus around them^24^ and that other jurisdictions and individuals have preferred terms for similar roles and processes (eg, patient and public involvement, patient engagement, citizen participation).

## Methods

 Semi-structured interviews^25^ within a qualitative descriptive study approach^26^ were used to explore the experiences and beliefs of a range of Australian hospital stakeholders towards the use of social media as a tool for consumer engagement. An advisory committee of key stakeholders, including healthcare consumers and service providers, provided oversight of the research project.

###  Research Instrument Development 

 An interview guide exploring the experience of, beliefs about, and attitudes towards social media as a tool for consumer engagement in Australian public hospital service design and QI was developed in consultation with the advisory committee. Advisory committee members were involved in determining interview questions and testing the interview guide.

###  Data Collection 

 A convenience sampling method^27^ was used to recruit participants through the networks of the researchers and advisory committee, and via the communication channels and networks of Australian health organisations who consented to sharing the recruitment information.

 Eligibility criteria were: aged >18; living in Australia; experience in any of the following roles in an Australian public hospital: consumer representative, QI, consumer engagement/patient experience, communications; an interest in, or experience of, using social media (for any purpose); able to participate in a 60 minute interview (face-to-face, telephone or videoconference). Potential participants were given detailed information about the study and the opportunity to ask questions prior to completing an emailed consent form, which was signed and returned to the researchers prior to the interview being scheduled. Each participant’s consent was reconfirmed verbally at the start of their interview. Limited demographic data (age; gender; hospital role; state located; name and location of hospital) were also collected for each participant. Information about hospitals (size, service types, location, social media platforms used) was collected and compared with information publicly available on each hospital’s website.

 LW conducted all interviews with participants. Audio recordings were taken of all interviews and transcribed by LW. Interviews were conducted until data saturation was reached.^28^

###  Data Analysis

 A qualitative deductive content analysis method^29^ was used. An analysis framework was developed from the findings of the scoping review conducted by members of the author team.^11^ The analysis framework used to guide the first round of coding is provided in Supplementary file 1 (Table S1). While the majority of the analysis was conducted by LW, co-authors contributed to the analysis through discussions in meetings and via email to enhance trustworthiness of the data.^30^ At the start of the first round of coding, LW and NH tandem coded two interview transcripts to pilot the framework and to compare coding consistency. After the first round of coding, all authors participated in an in-depth group discussion of one of the interview transcripts to guide further analysis, which included identifying additional codes and themes. In subsequent rounds of coding conducted by LW, codes and themes were refined and regrouped, following the approach to deductive analysis described by Linneberg and Korsgaard.^31^ Data was stored and managed on NVivo 12.^32^

## Results

 Twenty-six semi-structured interviews were conducted between October 2019 and April 2020. Nineteen interviews were conducted via telephone, five via videoconference, and two face-to-face. The participants held roles across 18 Australian public hospitals. Key features of the participants are detailed in Table 1.

**Table 1:** Key Features of Participants

**Participant Code**	**Gender**	**Age Group**	**State**	**Metropolitan, Regional or Rural Location**	**Participant Role**
CE1	Female	46-55	Western Australia	Metro	Consumer engagement
CE2	Female	46-55	Victoria	Metro	Consumer engagement
CE3	Female	46-55	Victoria	Metro	Consumer engagement
CE4	Female	36-45	Queensland	Regional	Consumer engagement
CE5	Female	46-55	Queensland	Metro	Consumer engagement
CO1	Female	36-45	Victoria	Metro	Communications
CO2	Female	26-35	Victoria	Metro	Communications
CO3	Female	46-55	Queensland	Regional	Communications
CO4	Male	46-55	Victoria	Metro	Communications
CO5	Female	26-35	Victoria	Metro	Communications
CR2	Male	56-65	Victoria	Metro	Consumer representative
CR3	Female	56-65	Victoria	Metro	Consumer representative
CR4	Male	26-35	Queensland	Regional	Consumer representative
CR5	Female	56-65	Western Australia	Metro	Consumer representative
CR6	Male	66-75	Victoria	Metro	Consumer representative
CR7	Female	56-65	Queensland	Metro	Consumer representative
CR8	Male	66-75	Victoria	Metro	Consumer representative
CR9	Female	36-45	Queensland	Regional	Consumer representative
CR10	Male	66-75	Victoria	Metro	Consumer representative
CR11	Female	46-55	Queensland	Regional	Consumer representative
CR12	Female	18-25	Queensland	Metro	Consumer representative
CR13	Male	18-25	South Australia	Metro	Consumer representative
QI1	Female	46-55	Victoria	Metro	QI
QI2	Female	36-45	Victoria	Metro	QI
QI3	Female	36-45	Victoria	Metro	QI
QI4	Male	46-55	Victoria	Metro	QI

Abbreviation: QI, quality improvement.

###  Social Media Platforms Used

 Based on data gathered from the websites of the 18 health service settings, public social media profiles used by the hospitals for any purpose (not just service design/QI activities) included Facebook (n = 18), Twitter (n = 12), YouTube (n = 12), LinkedIn (n = 11), Instagram (n = 4), Patient Opinion (n = 1), and Vimeo (n = 1).

 Fourteen participants had direct experience of using social media as a tool for consumer engagement in service design and QI. Facebook (n = 11) was the most common platform participants had used. A full list of platforms used by participants for consumer engagement activities is presented in Table 2.

**Table 2:** Social Media Platforms Used by Participants for Consumer Engagement Activities

**Platform**	***n***
Facebook	11
LinkedIn	3
Discussion forums on organisational websites	2
Twitter	2
Bang the Table	1
Patient Opinion	1
Instagram	1
WhatsApp	1

###  The Use of Social Media as a Consumer Engagement Tool

 Participants with direct experience of using social media for consumer engagement in service design and QI had used social media to recruit participants to consumer representative roles or to consultation activities that occurred off social media platforms, as virtual spaces for consultation or co-design, and to seek public feedback on QI projects. Some participants also had experience of social media being used as a channel for complaints or compliments which could inform service design and QI activities. Further detail about how social media was used as a consumer engagement tool is published in a previous article from this study.^15^

###  Barriers to the Use of Social Media as a Tool for Consumer Engagement in Hospital Service Design and QI

 Four main barrier themes were identified – (1) *fears and concerns; *(2)* lack of skills and resources for social media engagement; *(3)* lack of organisational processes and support; *and(4)* problems with social media platforms and the changing social media landscape. *A summary of the barriers are included in Table 3.

**Table 3:** Summary of the Barriers and Enablers for the Use of Social Media as a Consumer Engagement Tool

**Barrier or Enabler? **	**Theme**	**Sub-themes**
Barriers	Fears and concerns	Fear, discomfort and/or a lack of confidence with using technology Concerns about the behaviour of others online Consumers reluctant to give negative feedback because of fears of repercussions from hospital or providers Organisational concerns about reputational damage
	Lack of skills and resources for social media engagement	Consumers unable to access hardware or internet, often due to a lack of money Lack of skills in using social media or hardware Lack of organisational resourcing (money, staff, time) for social media activities Organisations and providers having poor understanding of social media
	Lack of organisational processes and support	Service provider confusion about how feedback informs QI and service design activities Service providers concerned about added workload and extension of their role Access to social media sites through hospital devices blocked in some services Lack of effective communication between hospital teams Lack of organisational buy-in to the use of social media Social media accounts available at the regional health service level, but not the individual hospital level Reluctance by the hospital executive to resource functions outside of core medical business Perception of social media as a non-essential add on to consumer engagement activities Belief that current consumer engagement strategies are meeting hospital needs
	Problems with social media platforms and the changing social media landscape	Platforms constantly changing, new platforms coming online Written English-heavy nature of social media communications Poor handling/securing of private information by social media companies Disability access and usability issues with some platforms
Enablers	Hospitals facilitating access and use	Organisational policies, processes, guidelines and roles related to social media use Adequate resourcing of consumer engagement Consumers involved in the design of social media-based consumer engagement activities Train and support consumer and provider users in the use of social media Help consumers and providers access software and hardware for social media-based consumer engagement activities Organisational and executive buy-in for social media-based consumer engagement Learning from the experience of other organisations Social media being part of a suite of consumer engagement activities
	Making discussions safe	Monitoring and moderation of social media pages and groups Plans in place to manage and respond to negative comments/behaviours Ground rules and community standards for users, created and agreed upon by the users Moderators able to check in with users outside of social media groups Private groups/platforms used for engagement activities on topics that may be controversial or require people to share personal experiences Users have the option to be anonymous
	Cultivating a social media community	Promote social media pages through other hospital communication channels Content on hospital social media pages is consumer focused (not staff or organisation-focused) Hospitals encourage and support consumer-generated content Social media-based consumer engagement is targeted towards people who are already/more likely to be using social media Target audiences are asked if they want to use social media as an engagement method Some consumers are approached directly to be involved in social media-based engagement activities rather than relying on general recruitment call-outs alone Social media features such as polls, knowledge of algorithms and targeted advertising are used to engage target audiences Awareness and management of disability access issues on platforms Social media content developed is suitable for low English-literacy audiences Hospitals use multiple social media channels
	Building on success	Experiencing positive results from social media-based consumer engagement builds momentum for ongoing or future activities Share knowledge and experience gained through conducting social media-based consumer engagement Social media is used to publicly acknowledge and celebrate the contributions of consumers in service design and QI

Abbreviation: QI, quality improvement.

####  Fears and Concerns

 Almost all participants expressed their own, or others,’ fears and concerns about using social media as a tool for consumer engagement. These originated from both consumer and service provider users, and from hospital executives who had concerns for the organisation. Fears and concerns about social media use were perceived by participants to be important enough to stop, delay, or limit the use of social media as a consumer engagement tool within their hospital.

 Many interviewees had personal experience with consumer or service provider social media users who had fears, discomfort or a lack of confidence related to using information technology software and hardware.

 “*One of our older consumers, … she gives really good feedback, she’s quite wise, so she’s good value to have involved. … any sort of social media, she just says ‘I can’t do it, I can’t go there’. … for our community advisory committee, we talked about having an online platform for sharing the agendas and minutes and storing them, I think we were going to use Dropbox, she felt very distressed about that idea”* (CE2).

*“I saw it with both my mum, my parents and my grandparents, there was like a visceral fear with using a phone that wasn’t a landline. Even to touch the phone, it was like the phone was electrified or something*” (CR3).

 Most participants expressed concerns about poor behaviour of other people online, which could lead to bullying, harassment and privacy breaches. This meant that some people were reluctant to share personal information online, including their experiences and opinions.

 “*I know that I’m* … *less likely to post personal opinion on social media. So I’m not sure that you would get the best out of me through social media*” (QI4).

 The poor behaviour of other people was of particular concern if social media sites were not managed or moderated properly.

*“I think past experiences definitely shape the way people use social media. I know people included in groups before get really put off joining them again. I think when* … *platforms aren’t monitored or mediated, and there’s the opportunity for people to say mean things, and that not be taken down and no rules. I think people need structure and need rules to use these things”* (CR11).

 Additionally, some participants expressed that a barrier for consumer users engaging through social media was fear of being perceived as a ‘bad’ patient and experiencing poor care if they provided negative or critical feedback.

*“I think some of the fear would be*,* ‘if I write, and then I come up to the hospital, and they have my name* … *then what’s going to happen?’” *(CR10).

 Some participants also believed their hospitals had a culture of risk aversion. These participants thought their hospitals were unwilling to use social media because two-way communication with consumers in service design and QI activities made the organisation vulnerable to receiving negative feedback which could harm the hospital’s reputation. This was a particular concern when negative feedback could be posted on public social media sites.

*“…until 2016 we didn’t have social media*,* and that was because of the board at the time. They were nervous about the negativity I suppose, rather than seeing that it’s better to be open and somewhat transparent and have the conversation if you need to” *(CO3).

*“They’re definitely risk averse so everything that would go out would be very controlled I would imagine*” (CE1).

####  Lack of Skills and Resources for Social Media Engagement

 Almost all participants identified that a lack of skills around social media use, and resources to enable its use, were barriers to using social media as a consumer engagement tool.

 Consumer users can have difficulty accessing social media, often due to hardware and internet access issues. Lack of access could be due to consumers not having enough money to afford devices or internet access, or having unreliable internet connections at home.

*“A lot of people* … *that use [health service]* … *don’t have a smartphone* … *they find it hard to put aside even $50 or $100, $200 for a good smartphone*.* They might not have NBN [National Broadband Network] or a laptop computer or something either”* (CR3).

 A lack of skills in using social media was a barrier for both service provider and consumer users.

*“People* … *just don’t know how to use social media, and there are plenty of those. It’s a new skill for some people [learning] where to post, what to post, how to post, how the platforms work*,*those sort of things take time*” (CE1).

 Barriers due to skills and resourcing were also seen at the organisational level. A lack of organisational funding was a barrier for the use of social media as a tool for consumer engagement, which also created other resourcing barriers, such as a lack of staff or lack of staff time.

*“There’s a freeze on hiring at the moment. Not hospital wide, but certainly in the communications directorate* … *There is always a desire there to manage our social media communities in a more effective way*,* we’re doing well at the moment,* … *our engagement is growing, but there are definitely bigger steps that we need to take in terms of having * … *a community that delivers more for the hospital. And I think that requires more people”* (CO2).

 In terms of skills at the organisational level, some participants felt that hospitals were unskilled at using social media for two-way communication, and that social media was poorly understood by hospitals and service providers generally. This lack of skills and understanding was a barrier to hospitals using social media for consumer engagement.

*“I see the risks and I acknowledge them*,* but I think they lack knowledge on how you can create platforms that suit your needs. They don’t understand that you can close off certain features, they don’t understand that you can customise the experience for the user”* (CR11).

####  Lack of Organisational Processes and Support

 The majority of participants identified that a lack of organisational processes and support was a barrier for the use of social media for consumer engagement in service design and QI.

 There were a number of organisational barriers specific to service provider users. Some service provider participants expressed confusion around existing organisational processes around how feedback was used. The need for ethics approval to collect patient feedback in some services was a barrier to service design and QI activities generally, and there was added confusion about whether information gathered through social media (even if volunteered by consumers) could be used to inform QI activities under existing ethics arrangements.

*“I might be looking at one particular issue you get lots of feedback about that, but there’s all these other comments that are actually really good and fit with another project that you’re working on*,* but can you use that information in that setting? Because was this information given because we’re looking at this issue, not because we’re looking at that issue? Are they happy for us to take it into a different context and use it in a different way?” *(CE3).

 Service provider participants were also concerned about added workload or extension of their role if social media was introduced into consumer engagement activities.

*“I think they’d need to be reassured that it would be managed appropriately, and that’s it not going to add to their workload”* (CE5).

 In some hospitals, staff access to social media was blocked on hospital devices. As a result, staff using social media as part of their work sometimes needed to use their own devices and the hospital’s public Wi-Fi system.

*“We’ve got a lot of important privacy issues in terms of protecting our hospital system*.* And that comes at a cost because the firewalls are really locked down, and we have moved to [a] more open option because we now have a general Wi-Fi so everyone can connect through and send things. But using hospital devices to* … *use social media,* … *there’s still clunkiness to that. The work-around* … *is that people … are mostly using their own devices. So I’m using my own phone as my main tool for accessing Twitter, WhatsApp, all those things”* (QI3).

 At an organisation level the barriers were a lack of effective communication and collaboration between teams, and a lack of buy-in to the use of social media from the wider organisation. These barriers particularly affected the relationship between communications teams and the rest of the hospital.

*“As a communications professional and not a health worker I can’t be telling people what they should be doing*,* it all needs to be coming from our senior health staff. So I think that can potentially be another barrier if staff can’t be brought into social media and aren’t willing to engage with it”* (CO5).

 Some participants from rural and regional areas reported that organisational social media accounts were only created and managed at the regional health service level, rather than at the local hospital level. This limited their ability to engage consumers from their local community in local hospital service design and QI activities. This was a particular problem when the regional health service covered a large geographic area.

 Finally, a small number of participants described reluctance by the executive of their hospital to provide resources to any functions that were not seen as core medical business. Related to this, social media was sometimes perceived within organisations as an “add-on” tool rather than an essential part of a consumer engagement strategy, and some participants believed that there was a perception within their service that current consumer engagement strategies were adequate for hospital requirements.

*“And so, unless there is an occasion of service, it doesn’t have pre-eminence in their thinking of what’s valuable*.*So they’re not recognising the opportunity that fits around communication in their organisation, or how it could improve things” *(CE1).

####  Problems With Social Media Platforms and the Changing Social Media Landscape

 Some problems situated beyond the health service level, within social media platforms and the wider social media landscape, can act as barriers to using social media for consumer engagement in service design and QI. This theme reflects issues with social media platforms and the companies that run them, which are largely out of the control of consumers, service providers or hospitals.

 Constantly changing platforms and new platforms coming online meant that it was difficult for users to keep up with the technology, and impossible to be across every possible platform.

*“I grew up with computers* … *and I’ve seen the change *in *technology through the decades*.* I can’t keep up now it’s moving too fast for me, even when I talk about it, every five seconds there’s something different to do! I’m across the latest programs, but it’s hard to keep up, everything keeps changing*” (CE1).

 The written language-heavy nature of social media was seen as a barrier to engagement with consumer users who had difficulties reading English. Written social media communications were also a problem for both service provider and consumer users because of the lack of nonverbal cues, such as body language and tone of voice.

*“The challenge with it is how do you judge the content that’s coming back because* … *you don’t get the nuance that you get with body language and a conversation, even if it’s over the phone there’s body language or language in the spoken voice, you don’t get that on social media*” (QI4).

 Some participants distrusted how social media companies handle or secure private information. This was a barrier for both organisational and individual use of social media.

*“ So I guess that’s another barrier* … *there’s security concerns with WhatsApp, Dropbox, again there’s security concerns, we can’t really use it, we can’t use that*” (CE2).

*“I’ve got a consumer representative who we wanted to do a story on Facebook on* … *what she did to help with recruitment, and she said no, I’m not prepared to go on Facebook. So for some people it’s a lack of trust in the companies, all the various different companies*, … *that lack of trust around* … *what will happen with that information”* (CE3).

 Finally, some participants identified disability access and usability issues with some social media platforms, such as screen readers not working and problems with navigation or search functions. These issues could make using social media to engage with hospitals more difficult, or impossible, for some consumers.

###  Enablers for the Use of Social Media as a Tool for Consumer Engagement In Hospital Service Design and QI

 The enablers identified by participants were grouped into four themes: (1) *hospitals facilitating access and use*; (2) *making discussions safe*; (3) *cultivating a social media community*; and (4) *building on success. *A summary of the enablers are included in Table 3.

####  Hospitals Facilitating Access and Use

 Creation of organisational governance documentation and roles related to social media use was viewed by many participants as a way for hospitals to facilitate social media-based consumer engagement. Participants felt that hospitals should have dedicated social media roles, employees with social media expertise, and clear guidelines, policies and plans. Social media-based consumer engagement needed to be adequately resourced in terms of money, staff and time, and integrated into work plans where required.

*“Doing social media well is* … *a science and an art, and it won’t happen well with the best of intentions, it needs to be resourced around a particular strategy and a plan. But it does need to be resourced and you need* … *resourced expertise within a centre and then part of their job is to* … *support others to see the potential*”(CR7).

 Some participants felt that consumers should have a role in the design of social media-based consumer engagement activities within hospitals.

*“When we came up with our consumer participation plan earlier this year* … *one of the strategies we wanted to move towards was having* … *a client advisory group, and also parallel to that was* … *an online client panel where we could seek feedback from our clients in* … *other ways, not just face-to-face. And it [was] our client advisory group which ended up suggesting a platform where they could see each other’s responses*” (QI1).

 Hospitals could have a role in providing training and support around social media to build confidence and skills for service providers and consumers.

*“Probably training I guess. Understanding why we need to engage with consumers on social media. I guess training around the platforms too. Training around what’s sort of appropriate and what’s not*”(CO2).

 This support could extend to helping people access software and hardware if required for their role in a consumer engagement activity.

 Buy-in from hospital leadership and from the wider organisation was also seen by participants as an enabler for social media-based consumer engagement. They also identified that hospitals could learn from the experience of other organisations to help grow their own confidence and expertise in social media use.

 Finally, in terms of facilitating access to consumer engagement activities more broadly, participants believed that social media needed to be part of a larger suite of consumer engagement activities, which included face-to-face opportunities.

*“[Social media] was quite good at getting people’s feedback on things, getting people to talk about it. … it wouldn’t be the sole way of doing it, you still need to combine it with some interviews or phone conversations, it would be one tool you’d use if you were developing something*” (CE2).

####  Making Discussions Safe 

 Almost all interviewees believed that making discussions safe for all users was an important enabler for social media-based consumer engagement. Monitoring and moderation of social media pages was key to making discussions safe. Specific monitoring or moderation strategies discussed by participants were being prepared for negative comments and having plans to manage them, and having ground rules and community standards for users. Some interviewees recommended users be involved in creating ground rules and community standards.

*“You need somebody moderating or managing or keeping a close eye. What you’re trying to do is reduce the negativity. You want to be transparent, but at the same time you don’t want to accelerate negativity or incite that in that forum*”(CE5).

 Other monitoring and moderation strategies discussed were social media group managers having the ability to check in with users outside of groups if conversations were negative or potentially upsetting, being strategic or cautious about which topics or projects public social media pages were used to seek feedback on (eg, avoiding consulting on sensitive or controversial topics through public pages), and using private groups for engagement activities.

 Finally, participants acknowledged that having the option to be anonymous helped some consumers feel safe to engage with hospitals through social media. Anonymity could be an enabler for people who felt concerned about potential negative repercussions from their service providers if they shared feedback, or who wanted to share their story in a public forum without being identified as a user of the service.

*“So* … *people who do feel a bit vulnerable and who want to remain anonymous might be more inclined to use social media, so some vulnerable groups, who might be feeling vulnerable, might give that feedback*” (QI1).

####  Cultivating a Social Media Community

 Hospitals cultivating a large and well-functioning social media community that consumers could be drawn from for engagement activities was reported as an enabler for the success of social media-based consumer engagement. Participants believed it was important for hospitals to effectively promote their use of social media generally, and social media-based consumer engagement activities specifically, to build an audience and recruit to activities. The main strategy discussed by participants to attract followers to hospital social media pages was the hospital promoting its social media use through other hospital communication channels. Suitable communication channels mentioned by participants included the hospital website, hospital display boards and screens, and through service providers talking to consumers about the existence of hospital social media pages.

 Providing engaging, innovative and creative content on hospital social media pages was also seen as a way to build a social media following. Consumer-focused and user generated content were seen as particularly effective for attracting consumers to hospital social media pages.

*“…our group gave feedback that it was too focused on staff and we really didn’t have a connection to it as consumers. And from that feedback there’s been a big shift in what was produced. And I think there was an intranet Facebook page made for staff so they could still be updated about their side of it, but the public Facebook page became more community focused*” (CR10).

 To recruit to social media-based consumer engagement activities, participants recommended targeting engagement activities towards people who were already comfortable with using social media channels, seeking buy-in for social media engagement approaches from priority groups before deciding on an engagement strategy, and approaching people directly to be involved in social media-based engagement activities. These approaches should be used in addition to general recruitment callouts through social media.

 Participants also identified enablers around the design of social media pages and groups that could help hospitals grow their social media communities and improve their engagement activities. Managers of social media pages or groups could use social media applications and features – such as polls, algorithms, and targeted advertising – to reach their target audience and increase engagement. Platforms could be designed and built to be specific to user needs. Managers of social media pages also needed to be aware of disability access issues and create content that was suitable for low English-literacy audiences (eg, Simple English, audio-visual, and translated content). Using multiple channels for an engagement activity was also thought to increase engagement.

####  Building on Success

 Experiencing positive results from social media-based consumer engagement was viewed as an enabler for ongoing or future QI and service design activities.

*“I think that they just need to see a few strategies and a few case examples of how it has been effective and showing the results of speaking to a broad cross section of people*” (CO5).

 Some participants had opportunities to learn from another organisation’s use of social media for consumer engagement activities and found this valuable.

 Participants also felt that social media could be used to publicly acknowledge and celebrate the contributions of consumers in service design and QI. This could build momentum for future social media-based engagement activities within the hospital and in other organisations.

*“I’m very conscious about always tweeting about workshops we’re doing or ‘got this feedback from this patient about this thing’ or ‘this team have been working really hard and this is the thing they’ve changed’ and so I do a lot of that kind of work. Actively tweeting* … *And people really value that. It’s a great way to see that their efforts are recognised and valued and them to take pride in their work*” (QI3).

## Discussion

 This paper reported the platforms and methods used for engaging consumers via social media for QI and service design and explored perspectives and experiences of the barriers and enablers to effective use in the context of Australian public hospitals.

 The most popular social media platforms in Australia in 2020 were Facebook (89% of internet users), YouTube (54%), Instagram (45%), LinkedIn (20%), Pinterest (20%) and Twitter (20%).^33^ These platforms, and this order of popularity, were generally reflected in the platforms used by the hospitals included in this study. However, when the specific use of social media for consumer engagement activities was explored with participants, only 14 people had used social media for this purpose, and for most of them (n = 11) this was Facebook. The low numbers of participants who had experience with using other platforms for engagement indicates that even hospitals who are already using social media for consumer engagement might be missing opportunities to engage with consumers through platforms other than Facebook.

 The methods of social media use in consumer engagement described by participants in the current study confirm the previously developed typology of social media use for stakeholder engagement in health service change, design and QI activities.^11^ However, the ways in which Australian hospitals use social media for consumer engagement might be limited in comparison with other countries. Most notable was a lack of examples of hospitals seeking feedback from, or developing partnerships with, members of existing consumer-initiated and managed social media spaces. Leveraging existing social media patient and consumer communities for involvement in co-design or other hospital improvement activities is recognised as a potential benefit of health-related social media use.^10,34,35^ For example, in a study by Amann et al^36^ community managers in consumer-led online health communities allowed researchers, healthcare professionals and students to participate in the groups for the purpose of knowledge co-creation, particularly in regards to creating or improving services or products which benefited the community. This type of self-mobilised and consumer-initiated participation which occurs external to hospital or institutional social media spaces is sometimes referred to as community-controlled participation or citizen power.^37^ There is an opportunity for hospitals to increase and expand their use of social media for consumer engagement by building relationships with existing online consumer communities, while also recognising and respecting consumer control over decision-making and actions, which could challenge hospitals systems and ways of working.

 Healthcare organisations wanting to use social media will need to consider barriers and enablers for successful implementation. In summary, the barrier themes were (1) fears and concerns; (2) lack of skills and resources for social media engagement; (3) lack of organisational processes and support; and (4) problems with social media platforms and the changing social media landscape.The enabler themes were: (1) hospitals facilitating access and use; (2) making discussions safe; (3) cultivating a social media community; and (4) building on success.

 Many of the barriers and enablers around social media use identified by participants in this study are similar to those found in the broader consumer engagement literature. A lack of adequate resources, organisational support, skills and confidence have long been identified as barriers to consumer engagement in hospitals and health services internatonally.^38-40^ Similarly, a supportive organisational culture, good governance of consumer engagement activities, adequate resourcing, and training and support for service providers and consumers, are acknowledged enablers of consumer engagement.^38-40^ It is therefore important that hospitals and organisers of consumer engagement activities understand that social media-based engagement methods require the same good practices in terms of governance, planning, resourcing, and support as more typical engagement methods.

 Participants did identify barriers and enablers that were specific to social media-based consumer engagement. Fears about individuals being vulnerable to bullying, harassment and privacy breaches, and organisational concerns about damage to reputation, do not appear in the literature on face-to-face consumer engagement methods. Similarly, enablers which increase the safety of participants (such as monitoring and moderation, group rules) were seen as critical features of social media-based engagement but are less common in research on face-to-face consumer engagement. One notable exception is Aboriginal and Torres Strait Islander- and Māori-led health services research that has led the development of engagement processes that prioritise cultural safety.^41^ Given that unequal power dynamics are often inherent in consumer engagement activities,^13,42,43^ and fears have been expressed by consumers in this study that providing negative feedback might leave them vulnerable to a lower standard of care, it may be reasonable to assume that face-to-face engagement activities could also expose participants to unsafe situations such as bullying, harassment, discrimination and issues with privacy. Implementing strategies to enhance safety could be an important routine addition to all consumer engagement activities.

 Barriers and enablers around the use and accessibility of technology and social media were also unique to social media-based consumer engagement in comparison with the broader consumer engagement literature. For example, fears and concerns around the use of computers, mobile devices and social media were frequently reported. This is consistent with existing research around factors affecting uptake and use of information communication technologies (ICT), particularly in research with older people and those who are infrequent or non-users of ICT devices or social media.^44,45^ This barrier would need to be addressed by strategies which increase confidence with technology, such as training and ongoing support for both consumers and service providers. Additionally, there are barriers due to the social media landscape itself, such as the variety of platforms available, usability and accessibility issues of some platforms, and distrust of companies and handling of personal information. These may be more difficult to address, and it is essential that health services considering social media engagement strategies work with consumer representatives and target audiences to understand their specific barriers to social media use, and whether they can be reduced or overcome.

 Finally, it is important for implementers to understand that there might be some barriers that are not possible for individual services to easily overcome without supportive policy or infrastructure.^46^ Costs associated with social media use (including access to computers, devices, and internet) can be prohibitive for some people from low socioeconomic groups.^47^ There may be opportunities for hospitals and health services to advocate for affordable internet access to be considered a human right,^48^ or – on a more local level – provide access to free Wi-Fi or internet kiosks within the hospital. It is very unlikely, however, for hospitals to solve cost issues for every consumer wanting to participate in social media-based health service design or QI activities. To fully understand and address these issues it is important that hospitals consult with consumer groups to determine preferred engagement methods, channels or platforms, and continue to offer face-to-face and other non-social media-based consumer engagement methods in addition to social media-based initiatives. Additionally, given that consumer engagement in service design and improvement is enshrined in national healthcare accreditation standards,^23^ there may be a role for the Australian Government in supporting consumer access to hardware, software and internet to ensure equal opportunities for all consumers to participate in health service design and QI, regardless of their preferred method of participation.

###  Implementation Into Practice and Further Research

 The barriers and enablers identified by the participants in this study could be used as a guide for hospitals and health services wanting to incorporate social media-based consumer engagement into their service design and QI activities. However, as outlined above, it is important that health services also consider consumer engagement in their services more broadly, and seek to ensure engagement is well planned, appropriately resourced, well supported, and safe, no matter the methods used. The findings of this study could also help social media technology developers to understand how health service stakeholders use social media and the functions required to make platforms more suitable for consumer engagement activities.

 The outcomes and impacts of consumer engagement in hospital service design and QI generally are under-researched,^39,49^ as are the methods and outcomes of using social media as a consumer engagement tool.^11^ There are opportunities for hospitals and health services implementing social media-based consumer engagement activities to partner with researchers and build the evidence base around the methods and outcomes of social media as a consumer engagement tool in service design and QI activities. This may include comparative analysis of the differences between the experiences of service providers and consumers using social media to engage in health service design and QI to enable better tailoring of implementation strategies. Future research to better understand the particular barriers faced by people who are typically under-represented in consumer engagement activities,^50^ or at risk of low digital health literacy,^51^ could also benefit implementers who are targeting harder-to-reach communities as part of their engagement strategies. As indicated by participants in this study, sharing the methods and outcomes of research into engagement activities, particularly by publishing case studies, could also increase the uptake of social media-based consumer engagement by other hospitals and health services.

###  Limitations

 There were four main limitations of this study. The first was the use of a convenience sampling method rather than alternative method which may have allowed for more targeted sampling of people from communities in Australia who are known to be under-represented in consumer engagement activities^50^ and/or at risk of low digital health literacy.^51^ We also did not collect data from participants which would have allowed us to determine whether they belonged to at-risk communities. While some participants did discuss the experiences of at-risk groups and their use of social media for consumer engagement, and some participants may have identified as being part of these groups if we had collected that data, we may have been able to present more specific findings on the experiences of at-risk groups if we had used a different sampling method and more comprehensive data collection.

 The second limitation was that we recruited participants who were already familiar with using social media. While this use could be for any purpose (not just related to their hospital role), by interviewing people who were already inclined towards the use of social media some experiences or perceptions might have been missed – particularly around barriers to use.

 The third limitation was that twelve participants in the study did not have direct experience of the use of social media as a tool for consumer engagement in hospital service design and QI. As a result, their responses were largely speculative, rather than based in their own personal work experience. As a result, some barriers and enablers might have been missed.

 The final limitation was that most interviews were conducted before the coronavirus disease 2019 (COVID-19) pandemic was declared and restrictions on movement and gatherings of people were implemented in Australia. Since then, online working in hospitals has accelerated, and as a result some views and experiences expressed by participants in this study may have changed.

## Conclusion

 This study has shown that service providers and consumers consider it feasible, and potentially beneficial, to use social media for hospital service design and QI activities but future efforts should take account of barriers and enablers for meaningful engagement. Hospitals and their executives can overcome many of the identified barriers through developing good governance structures and clear documentation around social media use (policies, guidelines, plans), adequately resourcing social media-based activities, and providing training and support for both consumer and service provider users. Consumers should be involved during planning and delivery of social media-based engagement activities to ensure that strategies are suitable for different communities. For some consumers and service providers, barriers such as a lack of resources and distrust of social media companies could mean that social media-based engagement is less likely to be acceptable for them. Because of this it is important that hospitals continue to provide other methods of engagement (eg, face-to-face) alongside social media-based engagement strategies. Finally, there are opportunities for researchers to partner with health services implementing social media-based consumer engagement to develop greater understanding around the barriers and enablers for use, share methods and outcomes of engagement, and enable better tailoring of implementation strategies, particularly for groups underrepresented in typical consumer engagement methods.

## Ethical issues

 Ethics approval for this study was given by the La Trobe University Human Research Ethics Committee, application ID HEC19427.

## Competing interests

 Authors declare that they have no competing interests.

## Authors’ contributions

 LW was involved in obtaining funding, conceiving and designing the study, collecting data, interpreting and analysing data, and drafting and revising the manuscript. NH was involved in conceiving and designing the study, interpreting and analysing data, and drafting and revising the manuscript. JH, NJ, CL, BMS, and SR were involved in conceiving and designing the study, interpreting and analysing data, and revising the manuscript. SH was involved in obtaining funding, conceiving and designing the study, interpreting and analysing data, and drafting and revising the manuscript.

## Funding

 This work was supported by the National Health and Medical Research Council through a Postgraduate Scholarship [grant number GNT1168409].

## Supplementary files

Supplementary file 1 contains Table S1.
