# Supplementary material for: Social Media as a Tool for Consumer Engagement in Hospital Quality Improvement and Service Design: Barriers and Enablers for Implementation
Source: Int J Health Policy Manag. 2021 Nov 3;11(10):2287–98. doi: 10.34172/ijhpm.2021.151 (PMC9808274; doi:10.34172/ijhpm.2021.151)
Supplement: Supplementary file 1 — contains Table S1. [file ijhpm-11-2287-s001.pdf]

Article title: Social Media as a Tool for Consumer Engagement in Hospital Quality Improvement and Service Design: Barriers and Enablers for Implementation

Journal name: International Journal of Health Policy and Management (IJHPM)

Authors' information: Louisa Walsh[1]*, Nerida Hyett[2], Jayne Howley[3], Nicole Juniper[4], Chi Li[5], Belinda MacLeod-Smith[6], Sophie Rodier[7], Sophie Hil[1]

[1]Centre for Health Communication and Participation, La Trobe University, Bundoora, VIC, Australia.

[2]La Trobe Rural Health School, La Trobe University, Bendigo, VIC, Australia.

[3]Consumer Representative, VIC, Australia.

[4]Orygen, Parkville, VIC, Australia.

[5]Albury Woonga Health, Wodonga, VIC, Australia.

[6]Safer Care Victoria, Melbourne, VIC, Australia.

[7]Northern Health, Bundoora, Australia.

(*Corresponding author: Louisa.walsh@latrobe.edu.au)

Supplementary file 1

Table S1: Analysis Framework

Barriers	Lack of access to and familiarity with social media	• Lack of resources and access<br>• Lack of skills and confidence in using social media<br>• Lack of familiarity with using social media for health or QI purposes
	Lack of organisational processes and support	• Concerns about implementation and evaluation processes<br>• Issues with organisational culture and lack of executive support
	Concerns about how people behave online	• Concerns about managing messages, people and interactions in a public forum<br>• Concerns about privacy and professional behaviour<br>• Unwillingness to share personal information online
	Problems with social media platforms	• Rapid changes in the social media environment<br>• Poor platform usability

Enablers	Making discussions safe	• Making discussions safe
	Facilitating access and use for all stakeholders	• Making use of social media easier for target audiences<br>• Organisational systems, processes, resourcing and partnership<br>• Making access to social media easier for all users<br>• Providing multiple ways to engage
	Providing high quality content and incentives	• Delivering engaging, trustworthy and targeted content<br>• Users gaining benefits from participation
	Building a social media community	• Fostering connections between users in a community<br>• Organisations promoting their use of social media<br>• Targeting people who are most likely to use social media
